# Sexual Function of Women with Infertility

**DOI:** 10.1055/s-0038-1673699

**Published:** 2018-10-31

**Authors:** Priscilla Bianchini Salomão, Paula Andrea Navarro, Adriana Peterson Mariano Salata Romão, Maria Rita Lerri, Lúcia Alves da Silva Lara

**Affiliations:** 1 School of Medicine, Universidade de São Paulo de Ribeirão Preto, Ribeirão Preto, SP, Brazil

**Keywords:** Marital Infertility, sexuality, female sexual dysfunction, anxiety, depression, assisted reproduction, infertilidade conjugal, sexualidade, disfunção sexual feminina, ansiedade, depressão, reprodução assistida

## Abstract

**Objective** To assess the sexual function, anxiety, and depression of infertile women relative to a control group.

**Methods** Infertile women (infertile group, IG) of reproductive age were invited to participate in this controlled study. A control group (CG) of women was recruited from the general population of the same city. Sexual function was assessed by the Female Sexual Function Index (FSFI), and anxiety and depression were measured by the Hospital Anxiety and Depression Scale (HADS).

**Results** A total of 280 women participated in the present study, 140 in the IG and 140 in the CG. The analysis of the FSFI scores showed that 47 women (33.57%) in the IG and 49 women (35%) in the CG had sexual dysfunction (FSFI ≤ 26.55; *p* = 0.90). Women with anxiety or depression had a greater risk of sexual dysfunction, and sexual dysfunction increased the risk of anxiety and depression. Married women had a lower risk of depression than single women who were living with their partners.

**Conclusion** Infertile women had no increased risk of sexual dysfunction relative to controls. Anxiety and depression increased the risk of sexual dysfunction in the studied population.

## Abstract (pt)

**Resumo:**

**Objetivo** Avaliar a função sexual, ansiedade e depressão de mulheres inférteis em relação a um grupo controle.

**Métodos** Mulheres inférteis (grupo infértil, GI) em idade reprodutiva foram convidadas a participar deste estudo. Um grupo controle (GC) de mulheres foi recrutado da população geral da mesma cidade. A função sexual foi avaliada pelo Índice de Função Sexual Feminina (FSFI, na sigla em inglês), e ansiedade e depressão foram medidas pela Escala Hospitalar de Ansiedade e Depressão (HADS, na sigla em inglês).

**Resultados** Um total de 280 mulheres participaram deste estudo, sendo 140 no GI e 140 no GC. A análise dos escores do FSFI mostrou que 47 mulheres (33,57%) no GI e 49 mulheres (35%) no GC apresentaram disfunção sexual (FSFI ≤ 26,55; *p* = 0,90). Mulheres com ansiedade ou depressão tiveram um risco maior de disfunção sexual e a disfunção sexual aumentava o risco de ansiedade e depressão. As mulheres casadas tiveram um risco menor de depressão do que as mulheres amasiadas.

**Conclusão** As mulheres inférteis não apresentaram risco aumentado de disfunção sexual em relação aos controles. Ansiedade e depressão aumentaram o risco de disfunção sexual na população estudada.

## Introduction

Infertility affects between 3.5 and 16.7% of the couples in developed countries and between 6.9and 9.3% of the couples in developing countries, but less than 25% of infertile people receive treatment.^1^ Assisted reproduction (AR) is an option for infertile couples. However, the procedures used to assess the cause of infertility and some AR techniques may involve invasive procedures and the use of drugs that can lead to hormonal changes that may compromise a woman's well-being, self-esteem,^2^ and sex life with her partner. Indeed, infertility is often associated with increased sexual dysfunction^3^ and with interpersonal difficulties in women with secondary infertility.^4^ Another study showed that the duration of infertility is associated with a high probability of sexual dysfunction in women.^5^ Infertility may cause emotional and/or sexual maladjustment for many reasons, such as social and familial pressure to conceive^6^ and loss of spontaneity in the expression of sexuality by the partners.^2^ On the other hand, there is evidence that women may feel more confident during the treatment for infertility and that these treatments may increase the intimacy with their partners.^7^ In developing countries, women may feel responsible for the infertility, and family planning seems to be the only issue for women.^8^ However, infertility is not only the responsibility of women. Infertility is a disease of the couple.^9^ Thus, there is conflicting evidence on the impact of infertility and of infertility treatments on the sex lives of couples.

The literature also shows that infertile individuals have a high risk of psychiatric disorders,^10^ and that these can adversely affect their physical and emotional health,^11^ lead to feelings of shame,^12^ trigger stress in the individual and in the relationship, and negatively impact the quality of life of the individual.^13^ These contradictory conclusions regarding the impact of infertility and of infertility treatment suggest that previous studies have not considered the multiple emotions associated with infertility, and also highlight that some additional factors may impact the sex lives of infertile couples.^7^ Thus, the present study aimed to assess the sexual function, anxiety, and depression of infertile women relative to a control group.

## Methods

**Fig. 1:**
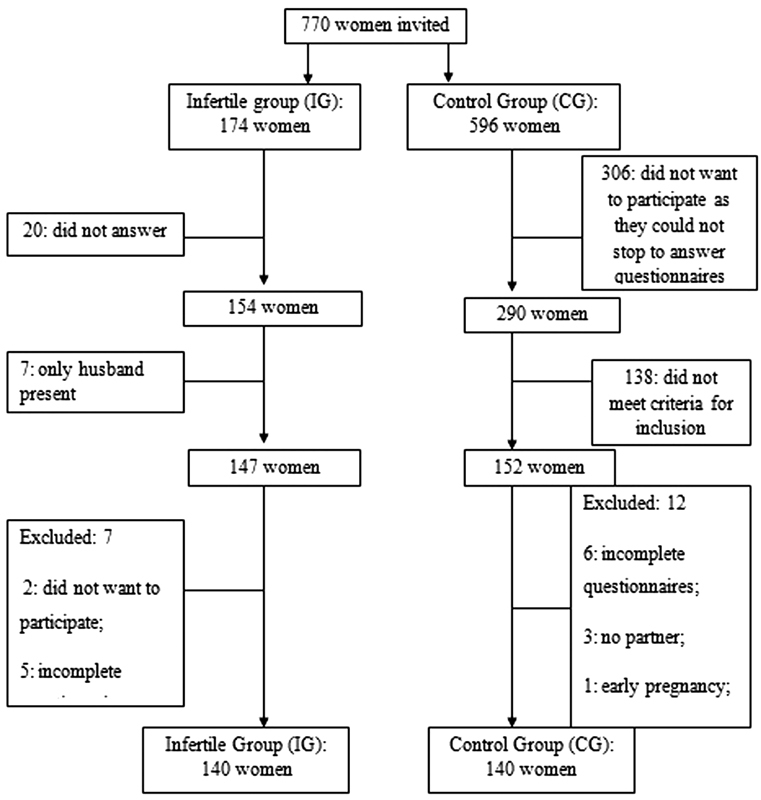
Recruitment of women in the infertile group (IG) and in the control group (CG).

The present case-control study examined sexual function in women of infertile couples. All women were in the reproductive period and undergoing treatment for infertility at the outpatient infertility clinic of a university center from July 2013 to April 2015. A psychologist in behavioral sciences (PBS) explained the content of the research to 174 consecutive infertile women when they were in the waiting room of the service and invited them to participate in the study. Ultimately, there were 140 infertile women in the infertile group (IG), and 140 women from the general population were in the control group (CG). Women in the CG had no diagnoses of infertility and were recruited while walking downtown (Fig. 1).

For recruitment of the CG, a PBS asked women for permission to explain the research. First, the researcher assessed eligibility by asking about age, presence in a stable relationship, and number of children. The aim of the research was explained to 596 women who were included in this preliminary screening. Three hundred and six women did not want to participate as they could not stop to answer questionnaires, 138 women did not meet the inclusion criteria, 6 provided incomplete answers, 3 were single, 1 was pregnant, 1 had 2 children, and 1 had more than 100 partners. None of the women in the CG were diagnosed with infertility and all were in stable relationships. Women in the CG were of reproductive age and had never been pregnant or had been pregnant once before. We have excluded women who were illiterate, pregnant, single, or had more than one child. The sample size was determined assuming a difference of 12% in the prevalence of sexual dysfunction between the groups, with a prevalence of 20% in the CG^14^ and of 8% in the IG.^15^ Our calculations indicated a sample size of 131 participants per group was needed, assuming a significance level of 5% and a test power of 80%. The IG included women of reproductive age, who were undergoing treatment for infertility and were in stable relationships with sexually active partners.

Clinical, anthropometric, and sociodemographic characteristics were collected through a semistructured questionnaire. The cause of infertility was determined from the medical records. The Hospital Anxiety and Depression Scale (HADS) was used to assess mood. This scale has 14 items and 2 subscales, with 7 questions regarding anxiety (HAD-A), and 7 regarding depression (HAD-D). There are 4 responses to each question (with a score between 0and 3), and the sum of the scores of each subscale provides a total score between 0 and 21. The cutoff score for anxiety was ≥ 8, and the cutoff score for depression was ≥ 9.^16^ Sexual function was evaluated with the Female Sexual Function Index (FSFI) and from data in the medical records of the participants. The FSFI is a questionnaire that has been validated for the population of the location where the study was conducted.^17^ It has 19 questions, with 6 subscales that assess desire, arousal, lubrication, orgasm, satisfaction, and pain. Each question is multiple choice and scored between 0 or 1 and 5. For the calculation of the total score (range: 2–36), the score of each subscale was multiplied by a factor, and the 6 scores were summed. A lower score corresponds to worse sexual function, and a score < 26.55 indicates sexual dysfunction.^18^

Women in the IG were invited to answer the FSFI and the HADS in a private room before medical evaluation. A psychologist stayed in the room as support if there were any questions. The CG also answered both questionnaires in the street where they were recruited by the psychologist. As the questionnaire is self-responding, the women of the CG received a support clipboard to keep the privacy of their answers. Women who reported changes in sexual function and expressed a desire for intervention by a sexual therapist, were referred to the Human Sexuality Studies Outpatient Clinic care. This project was approved by the Research Ethics Committee of our institution, and all participating women signed informed consent documents. All of the women signed the informed consent form.

Variables are provided in descriptive tables for quantitative and comparative analysis. The Fisher exact test was used to determine the significance of associations between variables of interest. For the comparison of quantitative variables between the groups, we have used the nonparametric Mann-Whitney test. To estimate crude and adjusted odds ratios (ORs), simple and multiple logistic regression were performed.^19^ The statistical analyses were performed using SAS software version 9.2 (SAS Institute Inc. Cary, NC, USA) and the PROC MEANS procedure. A *p*-*value* < 0.05 was considered significant.

## Results

Two hundred and eighty women participated in the present study, 140 in the IG and 140 in the CG (Table 1). In the IG, 104 women (74.29%) had primary infertility and 36 (25.71%) had secondary infertility. The cause of infertility was a female factor in 64 cases (45.71%), a male factor in 38 cases (27.73%), male and female factors in 35 cases (25.54%) and was unknown in 3 cases (2.1%). A total of 64 women (45.71%) had previously received in vitro fertilization (IVF) or intracytoplasmic sperm injection (ICSI).

**Table 1:** Anthropometric and clinical data of women in the infertile group (IG, *n* = 140) and in the control group (CG, *n* = 140)

Variable		IG *n* (%)	CG *n* (%)	*p-value*
Age (years)	< 40	130 (92.86)	125 (89.29)	0.40
	≥40	10 (7.14)	15 (10.71)	
Marital status	Living together	34 (24.29)	46 (32.86)	0.15
	Married	106 (75.71)	94 (67.14)	
Partner's age (years)	< 40	99 (70.71)	100 (71.43)	0.99
	≥40	41 (29.29)	40 (28.57)	
Time of relationship (years)	< 5	10 (7.14)	27 (19.29)	**< 0.01**
	≥5	130 (92.86)	113 (80.71)	
Schooling	Elementary	19 (16.38)	16 (11.43)	0.29
	High school	46 (39.66)	50 (35.72)	
	Higher education	51 (43.97)	74 (52.85)	
BMI (kg/m2)*	Low weight	2 (1.44)	2 (1.43)	0.72
	Normal weight	63 (45.32)	72 (51.43)	
	Overweight	44 (31.65)	42 (30)	
	Obese	30 (21.58)	24 (17.14)	
Sexarche	< 18 years	70 (50)	64 (45.71)	0.35
	≥18 years	70 (50)	76 (54.28)	
Frequency of intercourse	Every 2 months	1 (0.71)	1 (0.71)	0.41
	Once a month	0	3 (2.14)	
	Twice a month	4 (2.86)	1 (0.71)	
	Three times a month	3 (2.14)	2 (1.43)	
	1–3 days a week	111 (79.29)	108 (77.14)	
	4–6 days a week	21 (15)	24 (17.14)	
	Every day	0	1 (0.71)	
Number of partners	< 5	118 (84.28)	106 (75.71)	0.71
	≥5	16 (11.43)	33 (23.57)	
	Doesn't know	6 (4.28)	1 (0.71)	

Abbreviations: BMI, body mass index.

*Underweight: BMI < 18.5 kg/m2; normal weight: BMI = 18.5–24.9 kg/m2; overweight: BMI = 25–29.9 kg/m2; obese: BMI ≥ 30 kg/m2.

The IG had a higher median age (36 years [range, 32–38 years] versus 34 years in the CG [range, 31–37 years], *p* = 0.02) and longer duration of relationships (< 5 years: 7.14% in the IG versus 19.29% in the CG; > 5 years: 92.86% in the IG versus 80.71% in the CG, *p* < 0.01). However, the two groups had no significant differences in the number of women who were < 40 years old and > 40 years old (*p* = 0.40), in partners who were < 40 years old and > 40 years old (*p* = 0.99), marital status (*p* = 0.15), frequency of intercourse (*p* = 0.41), body mass index (BMI) with stratification by low weight, normal weight, overweight, and obese (*p* = 0.72), and education with stratification by elementary school, high school, and higher education (*p* = 0.29).

A FSFI score ≤ 26.55 was present in 47 women (33.57%) in the IG and in 49 women (35%) in the CG (*p* = 0.90). Table 2 shows the FSFI subscores of the women in both groups. The only significant difference was that women in the CG had a significantly higher subscore for excitation (*p* = 0.04) (Table 2).

**Table 2:** Subscores on the Female Sexual Function Index in the infertile group (IG, *n* = 140) and in the control group (CG, *n* = 140)

Variable	Group	Median (Q1-Q3)	p-value*
Desire	IG	3.60 (3.00–4.20)	0.51
	CG	3.60 (3.00–4.20)	
Excitation	IG	4.20 (3.60–4.80)	**0.04**
	CG	4.50 (3.60–5.10)	
Lubrication	IG	5.40 (4.20–6.00)	0.94
	CG	5.40 (4.20–6.00)	
Orgasm	IG	4.80 (4.00–5.60)	0.58
	CG	4.80 (4.00–5.60)	
Satisfaction	IG	5.20 (4.80–6.00)	0.52
	CG	5.60 (4.00–6.00)	
Pain	IG	5.60 (4.40–6.00)	0.90
	CG	5.60 (4.40–6.00)	

*Mann-Whitney *t*-test.

The analysis of the HADS scores indicated that 56 women (40%) in the IG and 51 women (36.42%) in the CG had anxiety (*p* = 0.62), and that 16 women in the IG (11.42%) and 18 women in the CG (12.86%) had depression (*p* = 0.86). However, considering the whole sample (*n* = 280), there were significant positive associations of sexual dysfunction (FSFI ≤ 26.55) with anxiety and with depression (*p* ≤ 0.01 for both comparisons) (Table 3).

**Table 3:** Association of sexual dysfunction (FSFI ≤ 26.55) with anxiety and depression among the entire sample (*n* = 280) according to the Hospital Anxiety and Depression Scale (HADS)

Variable	Sexual dysfunction		*p-value*
	Yes, *n* (%)	No, *n* (%)	
Anxiety (*n* = 107)	52 (54.2)	55 (29.9)	**< 0.01**
Depression (*n* = 34)	24 (25)	10 (5.4)	**< 0.01**

Abbreviation: FSFI, Female Sexual Function Index.

We have used multivariable analysis to identify the risk factors associated with sexual dysfunction with adjustment for age, BMI, marital status, length of relationship, education, pregnancy, contraception, parity, use of psychotherapy, cigarette smoking, alcohol consumption, age of partner, and risk of anxiety and depression (Table 4). The results show that women with anxiety or depression had a greater risk of sexual dysfunction (*p* < 0.01 for both) (Table 4).

**Table 4:** Logistic regression analysis of factors associated with sexual dysfunction among the entire sample (*n* = 280)

Variable	OR crude	95% CI crude	*p-value*	OR adjusted	95% CI adjusted	*p*-value
Age (≥40 years old *vs* < 40 years old)	1.03	0.40–2.67	0.95	1.09	0.37–3.21	0.88
BMI* (OW *vs* UW + N)	0.91	0.49–1.71	0.77	0.90	0.44–1.86	0.78
BMI* (O *vs* UW + N)	0.77	0.37–1.63	0.50	0.66	0.27–1.64	0.37
Marital status (married *vs* unmarried)	1.66	0.89–3.09	0.11	1.84	0.83–4.05	0.13
Time of relationship, years ( ≥5 *vs* < 5 years)	2.06	0.86–4.98	0.11	1.36	0.47–3.96	0.57
Schooling (high school *vs* elementary)	1.98	0.82–4.78	0.13	2.49	0.91–6.83	0.08
Schooling (complete high school *vs* complete elementary)	0.88	0.37–2.12	0.78	0.96	0.35–2.61	0.94
Pregnancy (yes *vs* no)	2.01	1.14–3.54	**0.02**	0.89	0.26–3.02	0.85
Contraception (yes *vs* no)	1.23	0.71–2.13	0.46	0.84	0.40–1.76	0.65
Parity (yes *vs* no)	1.82	1.05–3.17	**0.03**	1.79	0.46–6.98	0.40
Psychotherapy (yes *vs* no)	1.03	0.34–3.12	0.96	1.96	0.56–6.78	0.29
Cigarette smoking (yes *vs* no)	0.66	0.23–1.90	0.44	0.54	0.14–2.10	0.37
Alcohol (yes *vs* no)	2.04	0.40–10.47	0.39	3.31	0.45–24.23	0.24
Alcohol (social *vs* no)	0.92	0.51–1.67	0.78	1.08	0.52–2.22	0.84
Partner's age (≥40 *vs* < 40 years)	1.11	0.61–2.02	0.75	1.20	0.60–2.43	0.61
Risk of anxiety (yes *vs* no)	2.87	1.63–5.05	**< 0.01**	2.53	1.34–4.81	**< 0.01**
Risk of depression (yes *vs* no)	7.10	2.83–17.78	**< 0.01**	6.85	2.37–19.75	**< 0.01**
Group (case *vs* control)	0.77	0.44–1.36	0.37	0.86	0.35–2.09	0.73

Abbreviations: BMI, body mass index; CI, confidence interval; N, normal weight; O, obsese; OR, odds ratio; OW, overweight.

*Underweight: BMI < 18.5 kg/m2; normal weight: BMI = 18.5–24.9 kg/m2; overweight: BMI = 25–29.9 kg/m2; obese: BMI ≥ 30 kg/m2.

The adjustment for age, BMI, marital status, length of relationship, education, pregnancy, use of contraception, parity, use of psychotherapy, cigarette smoking, alcohol consumption, age of partner, and group (case versus control) indicated that women with anxiety had a greater risk of sexual dysfunction and depression (*p* < 0.01 for both) (Table 5).

**Table 5:** Logistic regression analysis of factors associated with anxiety among the entire sample (*n* = 280)

Variable	OR crude	95% CI crude	*p-value*	OR adjusted	95% CI adjusted	*p-value*
Age (≥40 years old *vs* < 40 years old)	0.79	0.31–2.03	0.62	0.81	0.29–2.27	0.68
BMI* (OW *vs* UW + N)	0.91	0.50–1.68	0.77	0.98	0.49–1.93	0.95
BMI* (O *vs* UW + N)	1.05	0.52–2.13	0.89	1.19	0.53–2.66	0.68
Marital Status (married *vs* unmarried)	1.33	0.74–2.38	0.34	1.47	0.72–3.00	0.29
Time of relationship years (5 or more *vs* <5)	1.59	0.72–3.51	0.25	1.25	0.50–3.11	0.63
Schooling (high school *vs* elementary)	1.02	0.44–2.35	0.97	0.90	0.35–2.30	0.82
Schooling (complete high scholl *vs* complete elementary)	1.06	0.47–2.39	0.88	1.17	0.47–2.91	0.73
Pregnancy (yes *vs* no)	1.06	0.62–1.80	0.83	1.62	0.51–5.13	0.41
Contraception (yes *vs* no)	0.82	0.48–1.39	0.46	0.85	0.43–1.68	0.64
Parity (yes *vs* no)	0.85	0.50–1.44	0.54	0.41	0.12–1.46	0.17
Psychotherapy (yes *vs* no)	0.79	0.26–2.40	0.68	0.81	0.24–2.73	0.73
Cigarette smoking (yes *vs* no)	1.08	0.42–2.75	0.87	1.05	0.35–3.16	0.93
Alcohol (yes *vs* no)	3.53	0.63–19.88	0.15	5.23	0.75–36.43	0.10
Alcohol (social *vs* no)	1.20	0.68–2.13	0.53	1.41	0.74–2.69	0.30
Partner's age (≥40 years old *vs* < 40 years old)	1.03	0.57–1.84	0.93	1.00	0.52–1.94	0.99
Sexual Dysfunction (yes *vs* no)	2.86	1.63–5.05	**< 001**	2.44	1.29–4.62	**< 0.01**
Risk of depression (yes *vs* no)	4.28	1.77–10.32	**< 0.01**	3.60	1.35–9.61	**0.01**
Group (case *vs* control)	1.23	0.72–2.11	0.46	1.01	0.45–2.27	0.98

Abbreviations: BMI, body mass index; CI, confidence interval; N, normal weight; O, obsese; OR, odds ratio; OW, overweight.

*Underweight: BMI < 18.5 kg/m2; normal weight: BMI = 18.5–24.9 kg/m2; overweight: BMI = 25–29.9 kg/m2; obese: BMI ≥ 30 kg/m2.

The adjustment for age, BMI, marital status, length of relationship, education, pregnancy, use of contraception, parity, use of psychotherapy, cigarette smoking, alcohol consumption, age of partner, and group (case versus control) indicated that women with depression had a greater risk of sexual dysfunction and anxiety, and that married women had a lower risk of depression than unmarried women (*p* < 0.01 for all) (Table 6).

**Table 6:** Logistic regression analysis of factors associated with depression among the entire sample (*n* = 280)

Variable	OR crude	95% CI crude	*p-value*	OR adjusted	95% CI adjusted	*p-value*
Age (≥40 years old vs < 40 years old)	0.33	0.04–2.55	0.29	0.27	0.03–2.62	0.26
BMI* (OW *vs* UW + N)	0.88	0.37–2.10	0.77	0.69	0.23–2.09	0.52
BMI* (O *vs* UW + N)	0.63	0.20–1.99	0.43	0.44	0.11–1.73	0.24
Marital Status (married *vs* unmarried)	0.52	0.24–1.16	0.11	0.21	0.07–0.60	**< 0.01**
Time of relationship, years (5 or more *vs* < 5)	2.31	0.52–10.17	0.27	2.37	0.43–12.97	0.32
Schooling (high school *vs* elementary)	1.02	0.34–3.09	0.97	0.53	0.14–1.96	0.34
Schooling (complete high scholl *vs* complete elementary)	0.53	0.17–1.66	0.27	0.53	0.13–2.09	0.36
Pregnancy (yes *vs* no)	2.82	1.20–6.63	0.02	4.61	0.87–24.36	0.07
Contraception (yes *vs* no)	1.50	0.69–3.26	0.31	1.31	0.46–3.67	0.61
Parity (yes *vs* No)	2.10	0.96–4.61	0.06	0.63	0.09–4.28	0.64
Cigarette smoking (yes *vs* no)	1.42	0.39–5.17	0.60	1.24	0.23–6.74	0.80
Partner's age (≥40 years old *vs* < 40 years old)	0.74	0.30–1.81	0.51	0.51	0.17–1.54	0.23
Sexual dysfunction (yes *vs* no)	7.62	3.11–18.69	**< 0.01**	7.59	2.72–21.14	**< 0.01**
Risk of anxiety (yes *vs* no)	4.14	1.80–9.51	**< 0.01**	3.51	1.36–9.06	**< 0.01**
Group (case *vs* control)	0.72	0.32–1.59	0.41	0.93	0.23–3.72	0.91

Abbreviations: BMI, body mass index; CI, confidence interval; N, normal weight; O, obsese; OR, odds ratio; OW, overweight.

*Underweight: BMI < 18.5 kg/m2; normal weight: BMI = 18.5–24.9 kg/m2; overweight: BMI = 25–29.9 kg/m2; obese: BMI ≥ 30 kg/m2.

We have also analyzed the effect of the cause of a couple's infertility on sexual dysfunction on the women in the IG. Twenty-seven women (19.70%) who were responsible for the couple's infertility had sexual dysfunction. This was significantly greater (*p* < 0.05) than when the male partner was responsible (*n* = 12; 8.76%), or when both partners were responsible (*n* = 8; 5.84%).

## Discussion

The present study aimed to evaluate the relationships of sexual function, anxiety, and depression in infertile women. The IG had a significantly greater median age than the CG; however, the two groups had similar proportions of women < 40 years old and > 40 years old. Moreover, the two groups had similar rates of sexual dysfunction after the adjustment for all confounding variables. It should be emphasized that the 2 year difference in the median age of the IG and of the CG is probably not clinically relevant because women in both groups were of reproductive age, had the same hormonal status, and therefore had the same clinical risks of sexual dysfunction.^20^ As previously demonstrated, sexual thoughts, sexual fantasies, and interest in sex are more common in younger women, and these decline with increasing age. In fact, females who are 40 years old have 25% fewer sexual fantasies those who are 25 years old.^21^ ^22^

There was a significant difference between the IG and the CG in the duration of the marital relationship. However, the multivariable regression analysis that controlled for confounding variables indicated that the duration of the marital relationship (< 5 years versus ≥5 years) was unrelated to sexual dysfunction, anxiety, or depression. It should be highlighted that there are no universal criteria for defining the duration of a long-term relationship, and previous studies have used from 2 to 10 years as criteria. A recent study used a cutoff of 2 years to define a long-term relationship and found that compatibility and duration of the relationship were positively associated with women seeking sexual intercourse.^23^ For each unit increase in a compatibility score, there was a 1.45-fold increase in the likelihood that a woman would have initiative to engage in sex, and those who were in a relationship for more than 2 years were 2 times more likely to have sexual drive. In contrast, men in long-term relationships had less sexual drive than they did at the beginning of their relationships.^24^ Another research indicated that a long-term marital relationship (> 10 years) was associated with reduced sexual desire,^24^ less intimacy, reduced arousal, and more sexual dissatisfaction.^25^

The present study indicated that the IG and the CG had similar proportions of women with FSFI score ≤ 26.55 (the threshold for sexual dysfunction). Our results may be compared with those of an Iranian study that compared fertile and infertile women. This previous study showed a significant difference between these groups in mean age and marriage duration, and that the infertile women had FSFI scores significantly lower than the fertile women. However, the age of the women in this Iranian study ranged from 15 to 70 years old, one-third of the sample was > 35 years old,^26^ and older women have a greater risk for sexual dysfunction. Our results corroborate a previous study of the effects of infertility on sexual dysfunction on women from Turkey. However, the control group in this study was from a gynecology outpatient clinic, and this group may be prone to more sexual complaints.^5^ The prevalence of sexual dysfunction in our study was similar to that in a previous case-control study^27^ as well as to that of a cross-sectional study, in which 35.6% of infertile women had FSFI scores < 26.55.^28^ On the other hand, another cross-sectional study found that 87.1% of the infertile women had sexual dysfunction.^29^ However, our study had a different design and sample size than these other studies. We speculate that our finding that infertility had no effect on sexual function could be due to the high resilience to psychological stress of our infertile couples, most of whom were very young.^30^ This hypothesis must be tested in future studies.

The IG had a significantly lower FSFI subscore for arousal than the CG. These results are consistent with a case-control study that showed a lower score in arousal of infertile women,^27^ although this study also reported that infertile women had lower scores in sexual desire.

We have found that sexual dysfunction increased the risk of anxiety and depression, and that anxiety and depression increased the risk of sexual dysfunction. Previous research indicated that infertile women are more likely to present with anxiety, low self-esteem, misperception of body image, fear of rejection, and sexual problems.^27^ Also, infertile women have an increased risk of psychological disorders, such as anxiety and depression,^10^ and this could affect their emotional and physical health,^11^ and may lead to feelings of shame because of the impossibility of conception.^12^

A limitation of the present study is that the IG and the CG were significantly different in marital status. Marriage has been recognized as a social institution since ancient times, so unmarried couples may feel “guilty” because of their status. Nevertheless, previous research showed that the number of years of infertility treatment had no effect on marital satisfaction. Also, we have not performed stratification by duration of infertility or studied the effect of previous infertility treatments.

## Conclusion

In the present study, infertile women had the same risk of sexual dysfunction as women from the general population. Anxiety and depression increased the risk of sexual dysfunction, and sexual dysfunction increased the risk of depression and anxiety.
